# Molecular Insights for Optimizing T Cell Receptor Specificity Against Cancer

**DOI:** 10.3389/fimmu.2013.00154

**Published:** 2013-06-19

**Authors:** Michael Hebeisen, Susanne G. Oberle, Danilo Presotto, Daniel E. Speiser, Dietmar Zehn, Nathalie Rufer

**Affiliations:** ^1^Department of Oncology, Lausanne University Hospital Center (CHUV), University of Lausanne, Lausanne, Switzerland; ^2^Swiss Vaccine Research Institute, Epalinges, Switzerland; ^3^Division of Immunology and Allergy, Department of Medicine, Lausanne University Hospital (CHUV), Lausanne, Switzerland; ^4^Ludwig Center for Cancer Research, University of Lausanne, Lausanne, Switzerland

**Keywords:** cytotoxic T cells, TCR-affinity, melanoma, immunotherapy, TCR engineering, TCR signaling, T cell activating receptors, T cell inhibitory receptors

## Abstract

Cytotoxic CD8 T cells mediate immunity to pathogens and they are able to eliminate malignant cells. Immunity to viruses and bacteria primarily involves CD8 T cells bearing high affinity T cell receptors (TCRs), which are specific to pathogen-derived (non-self) antigens. Given the thorough elimination of high affinity self/tumor-antigen reactive T cells by central and peripheral tolerance mechanisms, anti-cancer immunity mostly depends on TCRs with intermediate-to-low affinity for self-antigens. Because of this, a promising novel therapeutic approach to increase the efficacy of tumor-reactive T cells is to engineer their TCRs, with the aim to enhance their binding kinetics to pMHC complexes, or to directly manipulate the TCR-signaling cascades. Such manipulations require a detailed knowledge on how pMHC-TCR and co-receptors binding kinetics impact the T cell response. In this review, we present the current knowledge in this field. We discuss future challenges in identifying and targeting the molecular mechanisms to enhance the function of natural or TCR-affinity optimized T cells, and we provide perspectives for the development of protective anti-tumor T cell responses.

## Quantitative Aspects of Antigen Recognition by CD8 T Lymphocytes

Cytotoxic CD8 T lymphocytes recognize through their T cell receptors (TCRs) an antigenic peptide that is presented by MHC class I molecules (peptide-MHC, pMHC) on the surface of an infected or transformed cell. TCR triggering activates in T cells a signaling cascade, which leads to the release of effector molecules and to the cytolytic elimination of the cell that stimulated the T cell. The efficiency of triggering a T cell response critically depends on how well a TCR binds to a stimulating pMHC complex and stronger interactions are thought to cause more vigorous T cell activation than weaker interactions (Stone et al., 2009; Zehn et al., 2009). The dissociation constant *K*_D_ is a physical parameter that is generally used to describe the strength with which a TCR binds to a given pMHC complex (Zehn et al., 2012) and to which we usually refer to as the affinity of TCR and pMHC interaction.

**Figure 1:**
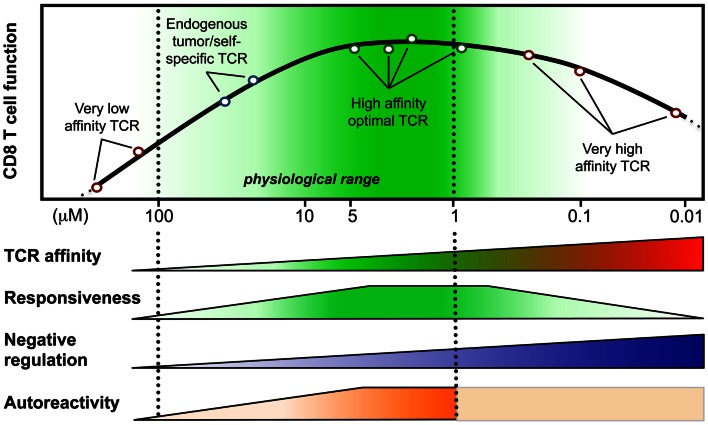
**Model integrating the relationship between T cell responsiveness (e.g., cell signaling, gene expression, and functionality) and TCR-affinity (in *K*_D_, μM) of human CD8 T lymphocytes engineered with anti-tumor TCR variants of optimized affinities (Irving et al., 2012; Hebeisen et al., 2013; Zhong et al., 2013)**. Optimal/maximal T cell effectiveness is observed with cells expressing affinities in the upper natural limit (*K*_D_ from 5 to 1 μM; dark green). Negative regulation mechanisms may counteract T cell responsiveness in T cells bearing very high affinities (depicted as blue gradients) (Corse et al., 2010; Slansky and Jordan, 2010; Hebeisen et al., 2013). Moreover, Zhong et al. (2013) recently described an affinity threshold (*K*_D_ around 10 μM) for maximal anti-tumor activity and autoreactivity (depicted as orange gradients).

Peripheral CD8 T cells express TCRs that only weakly react with self-peptide presenting pMHC and the *K*_D_ values of these interactions are in the range of 100–10 μM (Cole et al., 2007). In contrast, TCRs that interact with foreign-peptide presenting MHC with a *K*_D_ of up to 1 μM are frequently found among T cells that respond to pathogens (Davis et al., 1998). In fact, it is well established that immune responses to pathogen are dominated by cytotoxic T cells that express high affinity TCRs (Figure 1), and these cells are thought to be superior in executing effector function than low affinity T cells (Speiser et al., 1992; Alexander-Miller et al., 1996). Nonetheless, recent observations indicate that also a larger number of lower affinity T cell clones participate in immune responses. Moreover, it is well established that anti-tumor immune responses critically rely on lower affinity T cells, as most high affinity self/tumor-antigen specific T cells are usually thoroughly eliminated by both central and peripheral tolerance mechanisms. Within the subsequent sections, we will present key findings regarding the biology of cytotoxic CD8 T cells that respond with high or low affinity to antigen, we will describe how differences in affinity impact the outcome of a T cell response, and we will discuss several strategies to bypass the limitation that are linked to T cell responses mediated by low affinity T cells.

## Evidence for the Participation of Low Affinity CD8 T Cells in Immune Responses to Pathogens

To characterize how TCR–pMHC affinity impacts T cells in an infection, we expressed in pathogens a set of altered peptide ligands that gradually differ in the strength of binding to the OT-1 TCR. By infecting mice with pathogens expressing these ligands, we can mimic high, intermediate, or low affinity stimulation, as it would be the case with polyclonal cytotoxic T cells of which some respond with high and others with low affinity to pathogen-derived antigen (Zehn et al., 2009). Unexpectedly, we found that the OT-1 T cells initially responded similarly to pMHC complexes that very differently stimulated the OT-1 TCR. Even very low affinity complexes induced the same initial rapid T cell proliferation as high affinity ones. Low affinity-stimulated OT-1 CD8 T cells were early on phenotypically indistinguishable from cells stimulated by high affinity complexes. Expression of effector molecules such as granzyme B, as well as effector and memory T cell functions were surprisingly efficient (Zehn and Bevan, 2006; Enouz et al., 2012). It has also been shown that very low affinity stimulated T cells support pathogen elimination (Turner et al., 2008). Together, these findings indicate that lower affinity CD8 T cells fully participate in the immune response.

However, there is a major difference between low and high affinity CD8 T cells. Namely, the former undergo fewer rounds of division and decline in numbers faster than high affinity stimulated T cells. Thus, while undergoing full differentiation, low affinity primed effector T cells reach lower numbers. Therefore the high affinity T cells dominate in numbers at the time when T cell expansion is at its maximum.

Given their low numbers, one may question the importance of low affinity CD8 T cells. The large numbers of high affinity T cells at the peak of the immune response have so far distracted from exploring the relevance of low affinity T cells during infection. Several kinetic aspects may suggest that low affinity T cells could perhaps be more important than previously appreciated. In the naïve T cell repertoire, high affinity T cell clones specific to any given antigen are rare. In contrast, it is likely that low affinity T cell clones are more frequent. As low and high affinity clones expand equally at the beginning, there should be a larger number of low than high affinity effector T cells in the early phase of the T cell response, as we found in our experiments. The dominance of high affinity CD8 T cells develops later, because these cells overgrow the lower affinity T cells in the late T cell expansion phase (Zehn et al., 2009). Importantly, we noticed that low affinity T cells leave secondary lymphoid organs earlier than high affinity T cells, suggesting that the earliest wave of effector T cells that enter peripheral organs predominately consists of low affinity T cells. Thus, the critical early phase of pathogen elimination may be primarily achieved by low affinity cytolytic T cells (Zehn et al., 2009).

The number of low affinity T cells responding to one particular epitope is perhaps small. However, there could be many unknown epitopes recognized by low affinity T cells, which cumulatively might result in a reasonably sized T cell population. These considerations suggest that low affinity CD8 T cells play a more important role during infection than previously anticipated, which may have been underestimated in the past.

## Anti-Self and -Tumor Immune Responses are Frequently Mediated by Low Affinity CD8 T Cells

Anti-tumor immune response targets tumor-associated antigens such as cancer testis antigens (e.g., NY-ESO-1 or MAGEs) expressed by several tumors or differentiation antigens (e.g., Melan-A/MART-1, gp100, or tyrosinase) expressed in melanoma cells (Romero et al., 2002; Van Der Bruggen et al., 2002; Boon et al., 2006). Most of these antigens are expressed in the thymus (Kyewski and Klein, 2006) and accordingly, T cells with high affinity become negatively selected. As a backup, tumor-antigen reactive T cells can be eliminated in the periphery through mechanisms of peripheral tolerance (Kurts et al., 1997). However, it has been convincingly shown, that these mechanisms spare cytotoxic T cells that react with lower affinity to self- or tumor-antigens (von Herrath et al., 1994; Zehn and Bevan, 2006; McMahan and Slansky, 2007; Turner et al., 2008). Although, it is still often rather difficult to judge how effectively lower affinity CD8 T cells execute effector T cell functions, several strong line of evidence indicate that low affinity auto-reactive T cells are able to eliminate tumors and play a critical role in autoimmunity (von Herrath et al., 1994; Zehn and Bevan, 2006; McMahan and Slansky, 2007; Bulek et al., 2012). In fact, it becomes more and more clear that most self/tumor-specific cytotoxic T cells express low affinity TCRs and there is increasing evidence that the self/tumor-specific T cells are indeed capable to destroy cancer cells *in vivo* (Boon et al., 2006; Rosenberg et al., 2008). Moreover, it has been shown that self/tumor-antigen specific CD8 T cells can undergo considerable clonal expansion in cancer patients, differentiate to memory and effector cells, and persist during several years at relatively high frequencies (Speiser et al., 2011; Baitsch et al., 2012). These observations are well in line with the aforementioned findings that low affinity CD8 T cells participating in the response to pathogens may have great implications for anti-cancer immunity.

However, researchers must still deal with several challenges associated with activating low affinity CD8 T cells. For example, these cells require higher numbers of presented pMHC complexes than high affinity T cells before they become activated and for mounting an effector T cell response. Also, requirements for interactions with DCs by CD8 T cells of low TCR affinities are likely higher, to achieve sufficient TCR triggering and co-stimulation (Liechtenstein et al., 2012; Chen and Flies, 2013). Furthermore, lower affinity CD8 T cells undergo, as mentioned above, shorter clonal expansion following stimulation than high affinity T cells which means that fewer of such cells will be obtained following vaccination. Given these limitations, we need to find better ways to more effectively activate these T cells, to enhance their function, and to selectively interfere with the mechanisms, which prevent these cells from responding to tumors. One possible way to do this is to alter the kinetics with which the TCR of a tumor-specific T cell binds to its peptide-pMHC complex. Another approach would be to manipulate the signaling cascades downstream of the TCR.

## TCR-Affinity Optimization Against Cancer Antigens

Immunotherapy aims at mobilizing the body’s immune cells to fight against tumor cells in a highly specific manner. There are two biological strategies to achieve immune activity: active immunization with the use of vaccination and passive immunization. A form of passive immunotherapy is the adoptive cell transfer (ACT) of autologous T lymphocytes to patients with metastatic cancer (Restifo et al., 2012). This approach uses autologous TIL (tumor infiltrating lymphocytes), which are isolated from metastatic lesions, expanded *in vitro*, and selected for tumor reactivity. Remarkably, about 50 to 70% of patients with metastatic melanoma experience objective clinical responses, and up to 20% even have complete and durable responses (Rosenberg et al., 2011). Nevertheless, further improvements are necessary.

**Figure 2:**
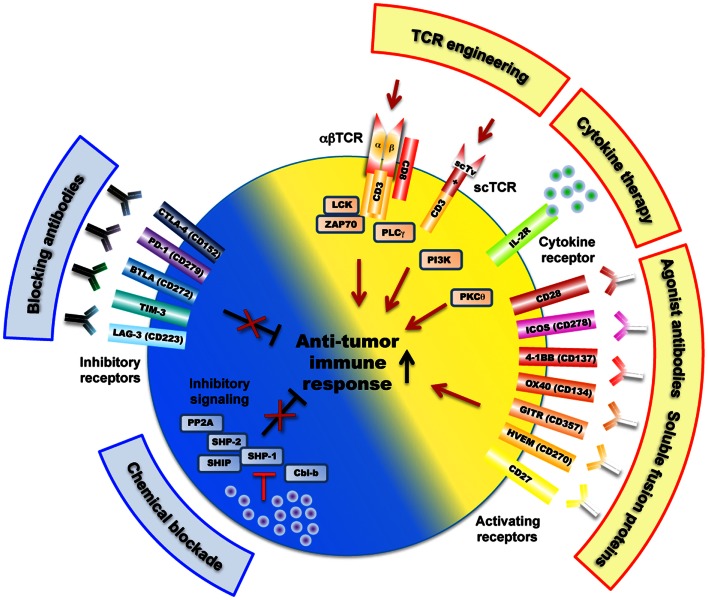
**Overview of mechanisms and potential therapeutic targets as a strategy to improve tumor-antigen reactive T lymphocytes**. These include a large variety of receptors (e.g., engineered TCRs, activating/inhibitory surface receptors, cytokine receptor) as well as TCR-downstream signaling molecules (e.g., SHP-1, SHP-2, PP2A) that regulate T cell activation, signaling, and function (e.g., killing, cytokine secretion) against cancer antigens. Of note, the scTv single VαVβ chain TCRs may be linked to intracellular signaling domains such as Lck and CD28, independently of the CD3 subunits (Aggen et al., 2012).

A limiting factor is the relatively low affinity of tumor-antigen reactive T cells. For improvement, T cells can be engineered with TCRs of increased affinity for tumor-antigens before transfer to patients (Figure 1). Indeed, this approach may augment the functional and protective capacity of tumor-antigen reactive CD8 T cells (Robbins et al., 2008, 2011; Bendle et al., 2009; Bowerman et al., 2009; Chervin et al., 2009; Johnson et al., 2009). In turn, TCR engineering also bears the risk that the normal tissue could be harmed. It has been demonstrated that T cells, whose TCR binds to pMHC complexes with very high affinities (*K*_D_ < 1 nM) lose antigen specificity and can become cross-reactive or allo-reactive (Holler et al., 2003; Zhao et al., 2007; Robbins et al., 2008). Importantly, genetically engineered T lymphocytes expressing very high affinity self/tumor-specific TCRs also target normal tissues expressing the cognate antigen (e.g., melanocytes in the skin, eye, and ear for Melan-A-specific T cells and neurons for MAGE-A3-specific T cells), and can mount harmful cytotoxic immune responses *in vivo* (Johnson et al., 2009; Morgan et al., 2013). Moreover, TCR mispairing between introduced and endogenous TCR α and β chains has also been shown to lead to off-target toxicity (Bendle et al., 2010; van Loenen et al., 2010). Therefore, TCR optimization through affinity alteration must include the evaluation of optimal T cell responsiveness and lack of cross-reactivity to ensure the safety of TCR-engineered T cells in clinical trials. Moreover, it must further include the development of new strategies to minimize the extent of mispairing (reviewed in Govers et al., 2010; Daniel-Meshulam et al., 2012), as elegantly shown by Aggen et al. (2012), describing the use of stabilized VαVβ single-chain TCRs (scTv; Figure 2). Unfortunately, unexpected auto-reactive responses may never be completely excluded. In that regard, it is important to further study the tissue distribution of self/tumor-antigen expression, to optimize the choice of antigens targeted by ACT therapy (e.g., cancer testis versus differentiation antigens) (Offringa, 2009).

## TCR-Affinity Threshold for Maximal Anti-Tumor CD8 T Cell Response

During recent years we established a panel of human CD8 T cells expressing engineered TCRs of optimized affinities against the tumor-antigen NY-ESO-1 presented in the context of HLA-A2. They were obtained through structure-based rational predictions (Zoete and Michielin, 2007; Zoete et al., 2010). The functional potential of these T cells (Schmid et al., 2010; Irving et al., 2012) showed that T cells expressing TCRs with affinities in the upper natural range (*K*_D_ from 5 to 1 μM) displayed greater biological responses when compared to those expressing intermediate affinity wild-type TCR (*K*_D_ at 21.4 μM) or very low affinity (*K*_D_ > 100 μM) (Figure 1). Unexpectedly, we noticed that T cells which express TCRs beyond the natural affinity range (*K*_D_ < 1 μM) showed a severe decline in their gene expression profile, signaling, and functionality (Irving et al., 2012; Hebeisen et al., 2013), despite retaining their antigen specificity and showing no broad cross-reactivity as observed in other studies (Holler et al., 2003). Major findings revealed that maximal T cell effectiveness was limited by at least two mechanisms (Figure 1). The first one was characterized by the preferential expression of the inhibitory receptor programed cell death-1 (PD-1) within T cells of the highest TCR affinities and this correlated in those cells with full functional recovery upon PD-1 ligand 1 (PD-L1) blockade (Hebeisen et al., 2013). The second one contrasted to PD-1 expression with the gradual upregulation of the Src homology 2 domain-containing phosphatase 1 (SHP-1) in CD8 T cells with increasing TCR affinities. Consequently, pharmacological inhibition allowed further incremental gaining of cell function in all engineered T cells, according to their TCR-binding affinities (Hebeisen et al., 2013).

Our observations provide new evidence that T cell activation and signaling may be limited to a given affinity threshold for the TCR-pMHC interaction and that above this threshold, T cells may not develop productive functions. They also nicely fit with other *in vitro* and *in vivo* studies that reported maximal T cell responses at an optimal TCR–pMHC off rate (*k*_off_) or *K*_D_ while functional attenuation was observed when kinetic parameters extended above the natural range (Kalergis et al., 2001; Gonzalez et al., 2005; McMahan et al., 2006; Carreno et al., 2007; Corse et al., 2010; Thomas et al., 2011). Furthermore, Krogsgaard and colleagues (Zhong et al., 2013) recently evaluated the TCR-affinity threshold defining the optimal balance between effective anti-tumor activity and autoimmunity *in vivo*, using human melanoma gp100_209-217_ – specific TCRs spanning within the physiological affinity range. Their results show the presence of an affinity threshold (around 10 μM) for maximal anti-tumoral activity and autoreactivity, suggesting that a relatively low-affinity threshold is necessary for the immune system to avoid self-damage (Zhong et al., 2013). Altogether, we and others propose that the rational design of improved self-specific TCRs for adoptive T cell therapy may not need to be optimized beyond the natural TCR-affinity range to achieve optimal T cell function and avoidance of unpredictable risk of cross-reactivity (Schmid et al., 2010; Slansky and Jordan, 2010).

Recently, Liddy et al. (2012) described the use of novel reagents termed immune-mobilizing monoclonal TCRs (or ImmTACs) against tumor-antigens including NY-ESO-1, which are fused to a humanized CD3-specific single-chain αβ fragment (scFv). These ImmTACs comprise TCRs of picomolar affinity range and allow to effectively redirect T cells to kill *in vivo* cancer cells expressing very low surface epitope densities. In line with previous studies from the same group (Li et al., 2005; Dunn et al., 2006), soluble monomeric TCRs possessing affinity ≈10^6^-fold higher than native TCRs showed a remarkable high degree of specificity for the cognate pMHC molecules. Possibly, soluble monomeric TCRs may allow circumventing the two major limitations associated with TCR engineering within CD8 T cells. First, the loss of target cell specificity associated with T cells expressing extremely high affinity TCRs (*K*_D_ < 1 nM) (Zhao et al., 2007; Robbins et al., 2008). And second, the functional defects of T cells with supraphysiological TCR affinities (*K*_D_ < 1 μM) (Kalergis et al., 2001; Gonzalez et al., 2005; McMahan et al., 2006; Corse et al., 2010; Thomas et al., 2011).

At present, what remains intriguing is how super affine TCRs modulate cell activation and responsiveness. One likely explanation is that in contrast to soluble TCRs, the cellular TCR expression integrates and potentiates the effect of several variables/parameters including TCR density, multivalent TCR clustering, and basal cell activation state (Stone et al., 2009). Furthermore, several observations including ours (Hebeisen et al., 2013) now indicate that T cell activation and signaling is also finely tuned by the proximal TCR-signaling complex as well as by activatory or inhibitory co-receptors, and will be discussed in detail below.

## Low and High Affinity Antigen Recognition Depends on the Proximal TCR-Signaling Complex

The TCR complex is composed of the TCR αβ chains, which are directly involved in the pMHC recognition, and of the invariant CD3 proteins, that contain in their cytosolic domains the immunoreceptor tyrosine-based activation motifs (ITAM) (Hedrick et al., 1984; Malissen et al., 1984; Letourneur and Klausner, 1992). TCR triggering elicits a series of membrane-associated events, leading to the transduction of signal across the plasma membrane and phosphorylation of key residues in the TCR-associated CD3 ITAM domains (Stefanova et al., 2003; James and Vale, 2012). Phosphorylation of CD3ζ-associated ITAM is mediated by the Src family kinases Lck and Fyn (Acuto et al., 2008) and form docking sites for several protein tyrosine kinases (PTKs) including the Syk-family kinase ζ-associated protein of 70 kDa, ZAP-70. Activation of ZAP-70 by Lck in turns results in phosphorylation and activation of other proteins and recruitment of adaptors (e.g., LAT and SLP-76). This initiates the formation of multi-molecular signalosomes, leading to the subsequent generation of secondary messengers and of multiple distal signaling cascades (Acuto et al., 2008; Smith-Garvin et al., 2009).

CD8 T cells may further adapt these signaling pathways to different stimulation conditions and different requirements for antigen sensitivity. Several lines of evidence indicate that differential patterns of CD3ζ ITAM phosphorylation directly modulate TCR-pMHC mediated downstream signaling and that ITAMs can act as both positive (ITAMs) and negative (inhibitory ITAMi) cell signaling regulators (Blank et al., 2009). For instance, resting peripheral T cells have a constitutive pattern of phosphorylated ITAMs, and incomplete CD3ζ ITAM phosphorylation after TCR triggering can by itself become inhibitory depending on the nature of the TCR ligand (Kersh et al., 1999). Thus, it is of great importance to further explore whether distinct CD3ζ ITAM phosphorylation states could also influence cell activation and responsiveness along the range of TCR-affinity and particularly in engineered CD8 T cells of supraphysiological affinity TCRs.

Lck represents another key regulatory element involved in the modulation of proximal TCR activation and signaling, and Lck activation stage may currently be viewed as a sensor of the strength of TCR engagement. On the one hand, weak binding of the TCR triggers Lck-dependent activation and recruitment of SHP-1, which in a classical feedback loop inactivates Lck and downregulates TCR signaling. On the other hand, stronger TCR activation induces an Erk-dependent Lck phosphorylation that impairs the inhibitory SHP-1 recruitment and in contrast reinforces TCR signaling by decreasing the threshold of T cell activation (Stefanova et al., 2003). Interestingly, as mentioned above, we recently used a panel of CD8 T cells engineered with TCRs of incremental affinities for an NY-ESO-1 derived peptide and saw that SHP-1 phosphatase was upregulated in a TCR-affinity-dependent manner, with the highest levels in T cells of the supraphysiological TCRs (Hebeisen et al., 2013). These observations further suggests that SHP-1 may play a dual role and restricts not only T cell signaling at the very low range of TCR stimulation (e.g., antagonist ligands) as described by Stefanova et al. (2003), but also at the higher range.

Other phosphatases have been shown to act on the proximal TCR signaling such as Lyp, a PTPN22 encoded phosphatase, and together with Csk inhibit T cell activation, likely through dephosphorylation of the activating tyrosine on Lck and ZAP-70 (Cloutier and Veillette, 1999). The importance of PTPN22 is highlighted by the observation that PTPN22 deficient mice have augmented TCR-induced phosphorylation and activation (Hasegawa et al., 2004). Furthermore, a point mutation in PTPN22 has been found associated with several autoimmune diseases (Mustelin et al., 2005). The precise role of PTPN22 in T cell activation remains unknown and there is contradictory data on the effect of the polymorphism found in autoimmune patients and whether or not it causes a loss or gain of function (Vang et al., 2005).

These TCR-affinity-dependent feedback mechanisms are likely part of a tunable instrument that enables T cells to adapt their reactivity to different stimulatory conditions, and we have just began to understand how those are achieved. For instance specific microRNAs such as miR-181a are thought to be critical in augmenting TCR-signaling sensitivity during positive selection in the thymus (Li et al., 2007). The expression of miR-181a has been shown to decrease the amount of several phosphatases, resulting in an elevated steady-state level of phosphorylated proteins of the TCR-signaling cascade and therefore a reduction in the TCR-signaling threshold (Li et al., 2007; Ebert et al., 2009). TCR activation and signaling transduction may also be negatively regulated by SHP-1 phosphatase and contributes to the settings of threshold during thymocyte selection (Plas et al., 1996; Acuto et al., 2008). Moreover, SHP-1 and SHP-2 can be recruited by multiple inhibitory surface receptors in T cells, and inhibit TCR signaling through dephosphorylation of proximal targets including Lck and ZAP-70 (Lorenz, 2009). In line with this concept, Yokosuka et al. (2012) recently showed that ITIM-containing PD-1 could directly inhibit TCR-mediated signaling by recruiting SHP-2 phosphatase in a TCR stimulation strength-dependent manner.

## Cytotoxic CD8 T Cell Responses are Regulated by Activating and Inhibitory Surface Receptors

Co-stimulatory and inhibitory membrane receptors have great influence on T cell responses (Chen and Flies, 2013). T cell co-stimulation prevents T cell anergy, a state of unresponsiveness that is induced after TCR stimulation in absence of co-stimulation (Figure 2). This was first observed when studying co-stimulation via CD28 that binds to its ligands B7.1 (CD80) and B7.2 (CD86) expressed on antigen-presenting cells (APC). This interaction also lowers the threshold for T cell activation, thus allowing increased IL-2 production and promoting cell proliferation and survival (Sharpe and Freeman, 2002). CD28 ligation stimulates T cell function by activating phosphoinositol-3-kinase (PI3K) and protein kinase C theta (PKCθ), and the downstream Akt, mTOR, and Ras signaling pathways, which eventually synergize with TCR signaling (Smith-Garvin et al., 2009). T cell activation also leads to surface expression of CTLA-4, which has a much higher binding avidity to B7.1 and B7.2, and thus outcompetes CD28 (Greene et al., 1996). Possibly, this may be the main reason for CTLA-4 mediated T cell inhibition. In addition, it has been shown that CTLA-4 directly triggers inhibitory signaling by interacting with SHP-1, SHP-2, and PP2A phosphatases, with the consequence of down-regulating TCR-signaling pathway (Scalapino and Daikh, 2008). CTLA-4 inhibition also occurs indirectly via retro-signaling through B7.1 and B7.2 and production of IDO in APCs (Grohmann et al., 2002) or by a process of trans-endocytosis of its ligands (B7.1 and B7.2) from APC (Qureshi et al., 2011). CTLA-4 may preferentially inhibit T cells with strong TCR signaling, as suggested by observations that accumulation of CTLA-4 at the immunological synapse depended on the strength of TCR triggering (Egen et al., 2002).

Programed death-1 is also highly upregulated in T cells following TCR stimulation, similarly to CTLA-4. Expression of PD-1 is not restricted to T cells, suggesting a broader role in immune regulation (Greenwald et al., 2005). PD-1 interacts with the two ligands PD-L1 and PD-L2, expressed non-redundantly in different tissues and cell types. CTLA-4-deficient mice have lymphoproliferative disorders and early fatal multi-organ tissue destruction (Tivol et al., 1995; Waterhouse et al., 1995), whereas PD-1-deficient mice spontaneously develop milder forms of autoimmune diseases (Nishimura et al., 2001). Based on the observed differential expression of CTLA-4 and PD-1 ligands, it is assumed that CTLA-4 plays a preferential role in limiting T cell function early during thymocyte development and in secondary lymphoid structures, whereas PD-1 may mediate inhibition in the periphery, for example in maintaining long-term peripheral tolerance to self-antigens by preventing activation of self-reactive T cells that have escaped negative selection (Fife and Pauken, 2011). TCR down-modulation through TCR/CD28 signaling transduction represents a fundamental process regulating the initial events of T cell activation. Recently, the interaction of PD-L1 on DCs and PD-1 on CD8 T cells has been shown to contribute to ligand-induced TCR down-modulation (Karwacz et al., 2011). Furthermore, interference with PD-L1/PD-1 signaling inhibited TCR down-modulation, leading to hyper-activated and proliferative CD8 T cells in an arthritis model (Karwacz et al., 2011).

In humans, a regulatory polymorphism in PD-1 is associated with susceptibility to systemic lupus erythematosus and multiple sclerosis (Prokunina et al., 2002; Kroner et al., 2005), while polymorphisms of the CTLA-4 have been linked to multiple autoimmune diseases including asthma, systemic lupus erythematosus, Graves’ disease, and autoimmune thyroid diseases (Kristiansen et al., 2000). The induction of PD-L1 ligand expression was observed in several tumor cells as a mechanism of cancer immune evasion (Schreiber et al., 2011). A specific polymorphism of CTLA-4 was found to be protective for autoimmune disease, but associated with risk of multiple types of cancer (Sun et al., 2009).

Members of the tumor necrosis factor receptor (TNFR) superfamily represent further important co-stimulatory molecules, mediating survival signals to T cells after initial CD28-B7 interactions (Acuto and Michel, 2003) (Figure 2). Multiple members of TNFR/TNF ligand pairs have been shown to directly impact T cell function following TCR activation, namely OX40/OX40L, 4-1BB/4-1BBL, GITR/GITRL, CD27/CD70, and CD30/CD30L (Watts, 2005). These receptors and their ligands are expressed on a variety of immune and non-immune cells and are inducible and non-ubiquitous, suggesting that they are involved in modulating and coordinating global immune responses (Croft, 2009). Intense translational and clinical research in this field aims at modulating T cell function in pathological settings such as autoimmunity and cancer (Figure 2). TNFR/TNF family member ligation often induces bi-directional activating signaling pathways in both the APC and the T cell. The recruitment of TNFR-associated factors (TRAF) activate the NF-κB signaling pathway and increase the expression of anti-apoptotic molecules, thus promoting the survival of CD4 and CD8 T cells (Croft, 2009). Like CD28, TNFR signaling can also synergize with the TCR pathway to promote cell cycle progression and cytokine production. Finally, ligation of OX40 and 4-1BB may concomitantly block the generation of inducible regulatory T cells (Tregs), and may inhibit their suppressive activity (So et al., 2008).

A particularly unique and interesting member of the TNFR superfamily is HVEM (Herpes virus entry molecule). It binds to the TNFR ligands LIGHT and lymphotoxin Ltα3, which are predominantly co-stimulatory and pro-inflammatory in T cells. Curiously, HVEM also binds to BTLA and CD160, which are structurally similar to PD-1 and CTLA-4 and transduce inhibitory signals, in part through recruitment of SHP-1 and SHP-2 phosphatases (Watanabe et al., 2003; Sedy et al., 2005). The individual effects of HVEM interaction with its different ligands are particularly complex to elucidate since both receptor and ligands can be expressed on the same T cell, as well as on other immune and epithelial cell types (Shui et al., 2011). *Hvem^−*/*−^* and *Btla^−*/*−^* T cells were found to be hyper-responsive to TCR stimulation *in vitro*. Furthermore, *Hvem^−*/*−^* and *Btla^−*/*−^* knockout mice had enhanced susceptibility to autoimmune diseases, suggesting a predominant inhibitory role in T cells during inflammatory conditions (Watanabe et al., 2003; Wang et al., 2005). BTLA was found to inhibit tumor-antigen specific cytotoxic T cells in melanoma patients (Derre et al., 2010). HVEM may also interact in *cis* with BTLA expressed by the same cell, likely interfering with HVEM activation by other ligands (Ware and Sedy, 2011). Therefore HVEM seems to mediate immune stimulation or inhibition in a switch-like, bi-directional, and context-dependent mode, suggesting that HVEM/LIGHT/CD160/BTLA interactions represent an important regulatory network for controlling immune responses.

Together, combined TCR and CD28/TNFR triggering primes CD8 T cells, followed by positive and negative regulation. The latter involves CTLA-4, PD-1, and BTLA. This highlights the intricate regulatory network that controls the immune system in health and disease (Figure 2). These mechanisms can be exploited therapeutically in patients with infectious or malignant diseases, as well as in autoimmunity and transplantation (Fife and Bluestone, 2008; del Rio et al., 2010).

## Activatory or Inhibitory T Cell Signals may be Targeted for Therapeutic Improvements of Cancer Therapies

Since cytotoxic CD8 T cells and T-helper type 1 [Th1] cells have the potential to eliminate cancer cells and to mediate long-term protection from disease (Sallusto et al., 2010), it is important to increase the functions of these anti-cancer T cells in cancer patients. As mentioned above, basic immunology characterized a number of interesting pathways that can be targeted to enhance the performance of tumor-specific CD8 T cells. Some approaches have already reached clinical application, but most still need to be tested in clinical trials. The therapy that seems most efficient for melanoma patients is the adoptive transfer of autologous tumor-antigen specific T cells (Rosenberg, 2011). Molecular modification of T cells before transfer may eventually increase the clinical efficacy, despite that this is currently not the case (Speiser, 2013). Several small-scale clinical studies suggested clinical usefulness of inserting TCRs (Rosenberg, 2011) or chimeric antigen receptors (Porter et al., 2011; Kochenderfer and Rosenberg, 2013). Hopefully, larger patient numbers will soon benefit thanks to steady improvements of these techniques (Thomas et al., 2010; Di Stasi et al., 2011; Linnemann et al., 2011; Ochi et al., 2011).

Not only antigen receptors but also co-receptors can be targeted therapeutically (Figure 2). Receptors that inhibit T cell functions are particularly attractive. Ipilimumab (Yervoy ®) is a monoclonal antibody that blocks the inhibitory receptor CTLA-4. It was recently approved for the treatment of metastatic melanoma, as it improves the clinical outcome, likely due to enhanced numbers and functions of tumor-specific T cells (Hodi et al., 2010). More recently, remarkable benefit for patients with advanced kidney cancer, non-small-cell lung cancer, and melanoma (Ribas, 2012) was demonstrated due to treatment with antibodies against PD-1 (Topalian et al., 2012) or its ligand PD-L1 (Brahmer et al., 2012). Likely, these results represent real therapeutic progress, despite significant toxicity linked to autoimmune reactions. Also, antibodies that block LAG-3, TIM-3, B7-H3, or B7-H4 are under development (Pardoll, 2012). Certainly, the clinical oncology landscape will change during the next years due to these novel approaches.

In addition to the targeting of cell surface receptors, intracellular mechanisms may be considered. In the complex signaling network downstream of the TCR, there are several possibilities. Interventions are for example possible at the level of E3 ligases (Hoyne, 2011) (Figure 2). As therapeutic targets, the SHP protein tyrosine phosphatases have been proposed (Irandoust et al., 2009). A member of a new class of SHP-1 inhibitors is the tyrosine phosphatase inhibitor-1 (TPI-1) that has been shown to inhibit the growth of transplanted tumor cells in mice together with enhanced cytokine production by T cells (Kundu et al., 2010). However, optimal targeting is challenged by the fact that SHP-1 and many other signal transducers are widely expressed. For example, hematopoietic tumors are suppressed by SHP-1 (Lopez-Ruiz et al., 2011), thus precluding this approach for such diseases. Therefore, novel drugs are needed that promote TCR signaling more specifically, suggesting a drug development similar to what is pursued for optimizing the well known tyrosine kinase inhibitors (De Roock et al., 2011; Goldstraw et al., 2011; Cascone and Heymach, 2012). In parallel to approaches targeting TCR pathways, further immune cells and functions can be supported therapeutically, such as e.g., B cells, adhesion- and homing-receptors, or cytokines (Scott et al., 2010; Miller and Rhoades, 2012; Nylander and Hafler, 2012).

Most likely, we are only at the beginning of understanding the enormous potential that is associated with the therapeutic approaches discussed here. Significant progress is yet to come, despite that immunotherapy has already become standard therapy for some cancer patients. Besides, antibodies blocking CTLA, anti-PD-1, and anti-PD-L1 mAb treatments and adoptive T cell therapy are promising. Novel therapies need to be improved and validated. Furthermore, it is important to learn predicting which therapy is most suitable for which patient. Potentially predictive parameters are the frequencies of tumor-reactive T cells, their ability to migrate to tumor sites, their affinity for antigen recognition, status of effector function, and presence of inhibitory regulatory circuits. More precise knowledge on correlates of protection, and immune monitoring techniques for their characterization in individual patients will support the progress of T cell based therapy against cancer.

## Conflict of Interest Statement

The authors declare that the research was conducted in the absence of any commercial or financial relationships that could be construed as a potential conflict of interest.
